# Influence of Medication on Fatigue Six Months after Stroke

**DOI:** 10.1155/2016/2410921

**Published:** 2016-06-19

**Authors:** Amélie Ponchel, Julien Labreuche, Stéphanie Bombois, Christine Delmaire, Régis Bordet, Hilde Hénon

**Affiliations:** ^1^Degenerative & Vascular Cognitive Disorders, Univ. Lille, INSERM U1171, 59000 Lille, France; ^2^Department of Medical Pharmacology, Univ. Lille, 59000 Lille, France; ^3^Department of Neurology, Lille University Hospital, 59000 Lille, France; ^4^Department of Statistics, EA 2694, Univ. Lille, 59000 Lille, France; ^5^Department of Neuroradiology, Lille University Hospital, 59000 Lille, France

## Abstract

Poststroke fatigue (PSF) is frequent and affects patients' quality of life. Medication use was hypothesized as being responsible for PSF. Our objective was to evaluate potential relationships between 6-month PSF and medication use at discharge and 6 months after an ischemic stroke. This study is part of STROKDEM, an ongoing longitudinal cohort study, whose main aim is to determine predictors of poststroke dementia. Patients were included within 72 hours after an ischemic stroke and followed up with standardized evaluations. Medication use 7 days and 6 months after stroke was rated, and polypharmacy was defined as the number of categories of treatments received by a patient. PSF was evaluated using the Chalder Fatigue Scale. Medical history, vascular risk factors, depression, anxiety, and sleep disturbances were evaluated. One hundred and fifty-three patients were included: 52.9% presented PSF. PSF at 6 months was not predicted by medication use at discharge nor associated with medication use at month 6. We found severity of PSF to be increased in patients with polypharmacy. Our results suggest that PSF is not a side effect of drugs use, which more reflects presence of disturbances frequently observed after stroke such as depression, anxiety, or sleep disturbances. Clinical study is registered on clinicaltrials.gov (NCT01330160).

## 1. Introduction

After stroke, 16 [1] to 74% [2] of patients report experiencing pathological fatigue, defined as “a feeling of early exhaustion, weariness, and aversion to effort” [3]. Poststroke fatigue (PSF) was found to be associated with poorer quality of life [2, 4–8] and to have a negative impact on recovery during rehabilitation [9] and return to work [10]. It was also found to be associated with lower functional outcomes [4, 11, 12] and increased mortality [11, 13–15]. A better understanding of PSF could permit developing effective therapeutic approaches, which are still lacking [16].

In the last update of practice guidelines from the Canadian Stroke Best Practices Committees [17], experts advised that “PSF should be screened for common and treatable poststroke co-morbidities and for medications that are associated with and/or exacerbate fatigue.” In fact, fatigue is a potential side effect of various drugs [18] and a significant association between fatigue and medication use has been reported in the elderly [19]. Moreover, stroke patients often blame their medications as potential causes of PSF [20], leading to the question of a potential relationship between medications and fatigue complaints after stroke [21, 22].

In a recent systematic review of the literature, we found that medication use could be related to PSF [23]. PSF was found to be more frequent in patients receiving antidepressants [4, 24–26]. However, in three of the four published studies there was no adjustment on depression, which might be an important confounding factor. In the same way, PSF was found to be more frequent in patients receiving analgesics but without adjustment on pain [11, 24] and in patients receiving anxiolytics but without adjustment on anxiety [27]. Other conflicting results were obtained for sleeping pills [11, 28, 29] and antihypertensive drugs [25, 28, 30, 31]. PSF did not seem to be associated with use of lipid lowering drugs [25, 28, 31], beta-blockers [31], antiacids [25], antiplatelets, and anticoagulants [25, 28].

Thus, few studies have evaluated the potential influence of medications on PSF and their results are inconclusive [23], leading to the need of a more specific assessment of potential associations between medications and PSF, with a controlled follow-up period. The aim of the present study was to evaluate potential relationship between medication use and PSF 6 months after an ischemic stroke.

## 2. Methods

The present study is a prespecified ancillary study of the STROKDEM project, which is an ongoing observational multicenter hospital-based prospective cohort study. The main aim of STROKDEM is to identify prognostic factors of the onset of dementia or cognitive decline following acute stroke.

This study procedure was approved by the local ethics committee and registered on clinicaltrials.gov (NCT01330160). Patients met a clinical research associate in a face-to-face interview. After explaining the aims of this study, approval was obtained and participants gave written informed content.

### 2.1. STROKDEM Cohort

In the STROKDEM cohort, patients aged ≥ 18 years with no dementia and admitted for a stroke caused by ischemia or hemorrhage (with brain lesion on magnetic resonance imaging (MRI) performed at patient's admission) within 72 hours of symptom onset are consecutively included after providing written informed consent. Exclusion criteria are (i) prestroke dementia (defined as Informant Questionnaire on Cognitive Decline in the Elderly (IQCODE, [32]) summed score of more than 64), (ii) malformed, traumatic, pure-meningeal, or intraventricular hemorrhage, (iii) patient being under legal care of guardianship, (iv) contraindication for an MRI, (v) inability to speak and understand French language, and (vi) neurological deficits including aphasia severe enough to impact questionnaires and tests understanding.

Upon inclusion, data concerning sociodemographic characteristics, vascular risk factors, and medical history are collected after a face-to-face interview using a structured questionnaire. Clinical severity of stroke is assessed according to the National Institute of Health Stroke Scale (NIHSS) [33]. Etiological (imaging, vascular, and cardiac examination) data of the stroke, as well as blood samples to be used to run the pertinent biomarkers of poststroke dementia, are collected. All patients are planned to be followed up at 6, 12, 36, and 60 months. At each of these visits, evaluation of severity of neurological deficits (NIHSS [33]), presence of emotional disorders, fatigue, and functional status is performed according to standardized questionnaires [34–38]. Information about changes in treatment is collected using a structured questionnaire. At each follow-up visit, patients undergo a battery of neuropsychological tests, a cerebral MRI on a 3-Tesla system, and a drawing of blood samples intended for follow-up on the progression of the biomarkers.

### 2.2. Study on Poststroke Fatigue

**Figure 1:**
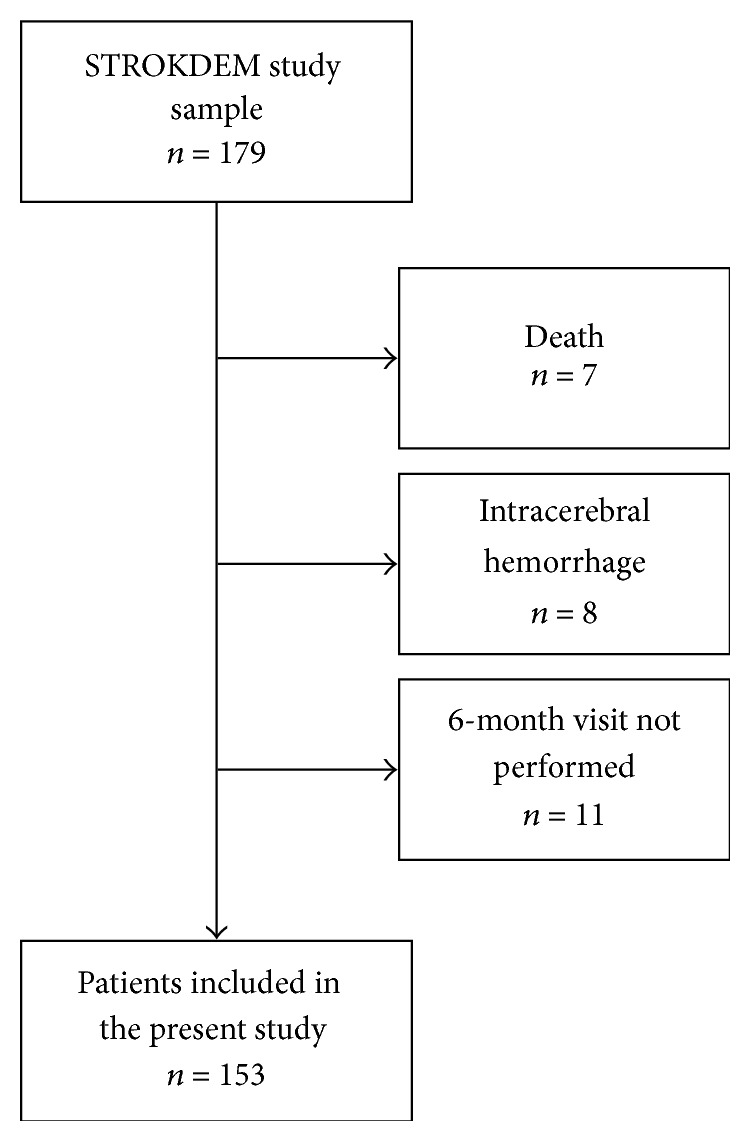
Flowchart of the study.

In this ancillary study on PSF, we included from the STROKDEM cohort the first 153 patients with an ischemic stroke admitted in the Stroke Unit of the Lille University Hospital, who had undergone their 6-month visit on July 2015 (see flowchart in Figure 1). For the purpose of our study we have collected the following data extracted from the STROKDEM database: demographic data (age and gender), vascular risk factors (arterial hypertension, diabetes mellitus, dyslipidemia, tobacco, and alcohol consumption), medical history with history of coronary heart disease, cancer, depression, and sleep apnea syndrome (based on polysomnographic records), previous stroke, or transient ischemic attack.

Stroke severity was assessed at the 6-month visit using the NIHSS [33]. We dichotomized the sample in two groups: patients without any focal deficit (NIHSS = 0) and patients with remaining focal deficit (NIHSS ≥ 1). Functional status was assessed using the Barthel Index (BI) [34]. Patients were divided in two groups: patients with complete recovery (BI = 100) and patients with residual functional deficit (BI < 100). Infarct location was assessed on MRI performed at the acute phase by a neuroradiologist blinded to clinical data and was defined as right hemispheric, left hemispheric, located in posterior fossa, or multiple.

Poststroke fatigue was assessed by trained vascular neurologists using the French version of the Chalder Fatigue Scale (CFS) [35]. This instrument is a multidimensional scale that evaluates fatigue among two dimensions: physical and mental fatigue. It is a self-report questionnaire with 11 items and a bimodal response system, with scores ranging from 0 to 11. Patients with a score of 4 or more were considered to have pathological fatigue [35, 39]. CFS scores were used as an ordinal variable to evaluate fatigue severity.

Patients underwent a systematic evaluation by trained neuropsychologists. Symptoms of depression were assessed with the Center for Epidemiologic Studies Depression Scale (CES-D) [36]. It is a 20-item self-rated questionnaire, with scores ranging from 0 to 60. Poststroke depression was defined as a score of 17 or more in men and 23 or more in women [40]. This scale was demonstrated to have good reliability and validity in stroke patients [41]. Anxiety was evaluated using the Hamilton Anxiety Scale (HAMA), a clinician-administered scale [37]. Scores ranged from 0 to 56, with a score over 6 defined as moderate to severe anxiety. This scale has good psychometric characteristics [42]. Score to item 4 of the HAMA [37] was used to evaluate sleep disturbances. This item covered difficulty in falling asleep, broken sleep, unsatisfying sleep and fatigue on waking, and dreams, nightmares, and night terrors. Patients who scored ≥1 to this item were considered as having sleep disturbances. New diagnosis of sleep apnea syndrome was also recorded. Patients with history or new diagnosis of sleep apnea syndrome were considered as having sleep apnea syndrome at 6 months.

Treatment use at hospital discharge (≤7 days after stroke onset) and current treatments 6 months after stroke were identified in a case report form using a standardized interview including antihypertensives (angiotensin-converting-enzyme inhibitors, angiotensin receptor blockers, calcium antagonists, alpha-blockers, and other central blood pressure medications), beta-blockers, antiplatelet drugs, anticoagulants, statins, antidiabetes medications, antidepressants, anxiolytics, and hypnotics. Each category of treatment was rated as 1 if used by the patient and 0 for nonusers, resulting in number of treatment categories ranging from 0 to 9. Polypharmacy was defined as the number of treatment categories received by one patient.

Other lipid lowering agents (fibrates, ezetimibe), anti-ischemic medications, nitrate derivatives, digoxin, antiarrhythmia medications, antipsychotic drugs, normothymic medications, antiepileptic medications, psychostimulant, hormone treatments, and others (allopurinol, vitamin E, omega 3, etc.) were also collected but not included in this study because they concerned less than 5 patients in the cohort.

### 2.3. Statistical Analysis

Quantitative variables are expressed as means ± standard deviation or medians (interquartile [IQR] or range), and categorical variables are expressed as numbers (percentage). Normality of distributions was assessed using histograms and Shapiro-Wilk test. Bivariate comparisons between patients with and without PSF were made using Student's* t*-tests (or Mann-Whitney *U* test for nonnormal distribution) for quantitative variables and Chi-Square tests (or Fisher's exact test when the expected cell frequency was <5) for categorical variables. Comparisons of medications at discharge and 6 months after stroke were made using McNemar tests. Associations of PSF with type and number of treatment categories used (at discharge and 6 months) were further adjusted for other patient's characteristics associated with PSF (at *p* < 0.10 in bivariate analyses) using logistic regression models. To avoid case deletion in multivariable analyses due to missing values on depression, anxiety, and sleep disturbances measurements scales (missing in 11 patients with PSF and 6 without), missing data were imputed to missing at random assumption by using regression switching approach (chained equation with *m* = 10 imputations obtained using the R statistical software version 3.03) [43]. Imputation procedure was performed using PSF and all variables listed in Table 1. Logistic regressions estimates obtained in the different imputed data sets were combined using Rubin's rules [44] and adjusted odds ratio (ORs) of PSF per each type and number of medications used were derived from these combined estimates.

Our first analyses were performed using CFS score as binary variable using a prespecified cut-off to define significant fatigue. Further analyses were performed by treating CFS score as an ordinal variable. Bivariate associations with CFS score were studied using Spearman's rank correlation coefficients for quantitative variables and using Mann-Whitney* U* tests for qualitative variables (all binary). Multivariate analyses were made using linear regression models on rank-transformed data.

Statistical testing was done at the two-tailed *α* level of 0.05. Data were analyzed using the SAS software version 9.3 (SAS Institute, Cary, NC).

## 3. Results

Demographic and clinical characteristics of the study population are detailed in Table 1. The mean age of the 153 included patients was 64 ± 13 years (range: 25 to 87) and 60.8% were men. One hundred and twenty-eight (83.7%) patients had a first-ever stroke and 97 (63.4%) had a NIHSS score of 0 at 6 months (median: 0; range: 0–11), corresponding to the absence of residual focal deficit. Six months after stroke onset, 32 (20.9%) were depressed, 64 (41.8%) were anxious, and 79 (51.6%) complained of sleep disturbances. Twelve (7.8%) had a sleep apnea syndrome.

Using a cut-off of CFS score of 4, 81 (52.9%) patients had 6-month PSF, with a median CFS score of 4 (IQR: 1–6). As shown in Table 1, patients with PSF were more frequently depressed (*p* = 0.025) and anxious (*p* = 0.003) and had more often sleep disturbances (*p* = 0.005) than patients without PSF. Nonsignificant between-group differences in gender, history of coronary heart disease, and 6-month BI score were noted.

The type and number of medications used at discharge and 6 months after stroke are described in Table 2 for overall study population and according to presence or not of PSF. Comparing medications at discharge and at 6 months, about 90% of patients received the same categories of drugs (see Table 3). Patients with PSF were more often treated with antidepressants (*p* = 0.034 at discharge and *p* = 0.007 at 6 months) and tended to receive more frequently beta-blockers at 6 months (*p* = 0.086). PSF was associated with polypharmacy (*p* = 0.024 at discharge and *p* = 0.020 at 6 months). After adjustment for between-group difference in demographic and clinical characteristics (i.e., gender, coronary heart disease history, 6-month BI score, depression, anxiety, and sleep disturbances), none of the medication use was associated with PSF, neither at discharge nor 6 months after stroke, with an adjusted OR (95% CI) of 2.13 (0.65–6.98) and 2.08 (0.70–6.14) for antidepressant use at discharge and 6 months and 1.73 (0.80–3.71) for beta-blockers at 6 months. Association between PSF and polypharmacy was not significant at discharge and at 6 months.

We performed additional analyses using the CFS score as an ordinal variable. Multivariate analyses showed a significant association between polypharmacy and CFS score and a tendency for a higher CFS score in patients receiving antidepressants and antihypertensive drugs (Table 4).

## 4. Discussion

In this study conducted in 153 consecutive patients with minor ischemic stroke, PSF at 6 months did not appear to be predicted by medication use at discharge nor associated with medication use at month 6. We found fatigue severity to be increased in patients with polypharmacy.

To the best of our knowledge, although many patients consider that the fatigue that they experience after stroke is a consequence of their treatment [20], this is the first study to have focused on the potential relationship between PSF and medication at discharge and 6 months after an ischemic stroke. In most previous studies on PSF, medications were not taken into account [23]. In the few studies on PSF that have taken medications into account, bivariate analyses were performed, without systematic adjustments on comorbidities.

Some data in the literature have suggested an association between antidepressant use and PSF [4, 24–26]. We also found antidepressant use to be more frequent in patients with PSF, but this association disappeared after adjustment on confounding variables (depression, anxiety, sleep disturbances, coronary heart disease history, gender, and BI score). This result is consistent with the results of a previous study [4] which reported a significant association between use of antidepressants and fatigue scores 3 months after an ischemic stroke (*n* = 218), which disappeared in a linear regression model taking into account depression, prestroke fatigue, and NIHSS score. This suggests that mood disorders and psychological distress could be more relevant to the explanation of PSF than the treatments which have been prescribed to treat these comorbidities. Moreover, fatigue is a symptom of depression, and although PSF is now distinguished as a specific syndrome [45], patients complaining of fatigue could easily be perceived as depressed, leading to more antidepressant use. Therefore, we cannot exclude that some patients receive antidepressant because of PSF. Medical teams should be advised to try to distinguish PSF from depression, as, until now, antidepressants have failed to show efficacy on PSF [46].

We did not find anxiolytics use to be more frequent in patients with PSF, which is in accordance with two separate studies having included, respectively, 220 [28] and 98 [25] patients from 1 to 27 months after stroke. Wang et al. (2014) [27] found use of benzodiazepines to be independently associated with PSF. However, they evaluated PSF at the acute phase of stroke (at days 13-14 after symptom onset). Moreover, although they took into account depression in their multivariate analysis, they did not consider anxiety. Yet, anxiety has been shown to be associated with PSF [23]. Additionally, Galligan et al. have suggested that stroke specific anxiety might be of equal importance to depression in terms of understanding PSF [25].

In our study, hypnotic use did not correlate with PSF, which is in accordance with a previous study which evaluated PSF in the acute phase of stroke [29]. By contrast, patients taking sleeping pills were more fatigued in the Bergen Stroke study in which PSF was assessed 6 to 24 months after stroke or transient ischemic attack [11, 24]. Nevertheless, in the Bergen Stroke study, there was no adjustment on the presence of sleep disorders.

We found a nonsignificant trend for an association between beta-blockers use at 6 months and PSF. As with antidepressants, this marginal association disappeared when taking into account confounding variables, in particular history of coronary heart disease. This is in accordance with a previous study which examined possible associations between beta-blockers use and PSF [31].

In our sample, neither statins nor antihypertensive and antidiabetes medications use was associated with PSF. Negative results were also observed for antiplatelets and anticoagulants. This replicates negative results found by others [25, 28, 31]. We did not find the trend observed in one study including 64 stroke survivors where the proportion of patients taking statins was higher in fatigued patients [31] even though fatigue is known as a side effect of statins [47, 48]. We did not replicate the results of a previous study suggesting use of antihypertensive drugs to be associated with PSF [30], where however only bivariate analysis was performed, without adjustment on potential confounding factors, and where no association was observed between PSF and the number of antihypertensive drugs taken.

Interestingly, polypharmacy was associated with more severe PSF at month 6. This could suggest that the more the comorbidities a patient has, the more likely he/she is to have severe PSF. Another hypothesis could however be that PSF could be partly induced by complex interactions which can occur when many drugs are administered.

There is to date no gold standard for the measurement of PSF [49]. We used the CFS because it is brief and easy to administer, which was important in our study where patients underwent multiple evaluations. This scale was also demonstrated to be well distinguished from mood evaluations, with a good sensitivity to change [50]. Moreover, this questionnaire has good psychometric properties and was demonstrated to be valid and reliable [35, 39, 51]. A cut-off of 4 is usually used to indicate significant fatigue and to differentiate between fatigue “cases” and “noncases” [35, 39]. This cut-off was used in various studies in chronic fatigue syndrome [52, 53], multiple sclerosis [54], pain [55], and stroke [9] as well as in general population studies [51, 56]. Using a cut-off of 4, we observed PSF in 52.9% of patients in our cohort. This frequency is quite similar to the 56% reported in a previous study using the same scale and cut-off [9]. This frequency is substantially above those reported in healthy young populations (10 to 18%) and older people (22%) [57, 58]. We however used CFS as an ordinal variable to evaluate potential relation between drug use and fatigue severity.

The major strength of our study is its prospective design and the use of standardized evaluations in face-to-face interviews, for the evaluation of PSF but also for the evaluation of mood and anxious disorders, which are important confounding variables, when evaluating the relationship between PSF and medication use, in particular antidepressant and anxiolytic drugs. This prospective design, with planned follow-up visits, also permits evaluating patients who are at the same delay from stroke. This may be of importance as frequency, and perhaps underlying mechanisms of PSF, can vary depending on the delay from stroke [59]. Another strength of this study was that PSF was assessed during neurological examination, which was separate from the neuropsychological evaluation assessing depression and anxiety. This study however has some limitations. First, our study population is not representative of stroke patients. The inclusion criteria used in the STROKDEM cohort had led to the inclusion of patients with mild neurological deficits. In fact, only one-third of our sample had residual focal deficits at 6 months. Therefore, results cannot be generalized to patients with more severe strokes. Second, our sample size was limited and this could partly explain negative results. We cannot exclude that some associations could have been overlooked due to the lack of adequate statistical power to detect small effects. This is particularly true for antidepressant and antihypertensive drugs: the association between presence of PSF and these categories of treatment was not significant but there was a tendency for more severe fatigue in patients receiving antidepressant or antihypertensive drugs (when analyzing CFS score as a continuous variable). In a posterior power calculation, we calculated the smallest significant between-group difference (expressed as effect size using odd ratio) that our study sample size (81 patients with PSF and 72 without) allowed us to detect with a 80% power. Assuming an exposure prevalence of 20% and 40% in patients without PSF, we could, respectively, detect an OR of 2.74 and 2.49. Third, due to a limited sample size of our cohort and the absence of conclusive evidence in the literature of an association between PSF and lesion site [23, 59], we decided to consider three groups for stroke location (right hemispheric, left hemispheric, and posterior fossa lesions). We did not find any relationship between stroke location and PSF. Moreover, evaluating the influence of stroke lesion on PSF at month 6 was not the scope of our study, whose aim was to evaluate potential relationship between PSF and medication use. There is, from our point of view, no background supporting the idea that this relationship could be modified by stroke location. Finally, in this study, fatigue was not evaluated before stroke and at the acute phase after stroke. As a consequence, we do not have data concerning the time at which fatigue has occurred. This leads to the impossibility to build up a real causality relationship between PSF and medication use. However, the STROKDEM study aims to assess long term cognitive and behavioral outcomes after stroke: our patients are currently followed-up (at 1, 3, and 5 years), which will allow an evaluation of the influence of treatments on persistent fatigue.

In conclusion, we did not find any significant relationship between PSF and any category of medication used at discharge and 6 months after stroke. This suggests that comorbidities, and in particular depression and anxiety, play a more important role than drugs in explaining PSF observed within the first months following stroke. Whether this finding will remain true when considering persistent fatigue remains to be evaluated and our patients are currently followed up with a planned 5-year follow-up period.

## Figures and Tables

**Table 1:** Demographic and clinical characteristics overall and according to 6-month poststroke fatigue.

	Overall (*n* = 153)	Poststroke fatigue		*p* value
		No (*n* = 72)	Yes (*n* = 81)	
*Demographic variables*				
Age, mean ± standard deviation	63.6 ± 12.8	62.3 ± 12.5	64.7 ± 13.1	0.24
Men	93 (60.8)	49 (68.1)	44 (54.3)	0.082

*Vascular risk factors*				
Hypertension	84 (54.9)	38 (52.8)	46 (56.8)	0.62
Diabetes mellitus	20 (13.1)	7 (9.7)	13 (16.0)	0.25
Dyslipidemia	72 (47.1)	30 (41.7)	42 (51.9)	0.21
Smoking	33 (21.6)	13 (18.1)	20 (24.7)	0.32
Alcohol consumption	15 (9.8)	5 (6.9)	10 (12.3)	0.26

*Medical history*				
Coronary heart disease	35 (22.9)	12 (16.7)	23 (28.4)	0.085
Previous stroke or transient ischemic attack	25 (16.3)	12 (16.7)	13 (16.0)	0.92
Cancer	14 (9.2)	9 (12.5)	5 (6.2)	0.18
Depression	13 (8.5)	8 (11.1)	5 (6.2)	0.27

*Stroke characteristics*				
6-month NIHSS, median	0 (0–11)	0 (0–6)	0 (0–11)	0.060
** ** *Score ≥ 1*	56 (36.6)	21 (29.2)	35 (43.3)	0.072
6-month Barthel Index score = 100 (complete recovery)	137 (89.5)	68 (94.4)	69 (85.2)	0.062
Infarct location				
** ** *Right*	62 (40.5)	34 (47.2)	28 (34.6)	0.24
** ** *Left*	64 (41.8)	24 (33.3)	40 (49.4)	
** ** *Posterior fossa*	21 (13.7)	11 (12.3)	10 (12.3)	
** ** *Multiple*	6 (3.9)	3 (4.2)	3 (3.7)	

*Emotional and sleep disturbances*				
Depression	32 (20.9)	10 (13.9)	22 (27.2)	0.025
Anxiety	64 (41.8)	22 (30.6)	42 (51.9)	0.003
Sleep disturbances	79 (51.6)	30 (41.7)	49 (60.5)	0.005
Sleep apnea syndrome	12 (7.8)	5 (6.9)	7 (8.6)	0.70

Values are expressed as number (%) unless otherwise indicated.

**Table 2:** Medication use at discharge and 6 months after stroke onset overall and according to 6-month poststroke fatigue.

	Overall (*n* = 153)	Poststroke fatigue		*p* value	OR^*∗*^ (95% CI)	*p* value^*∗*^
		No (*n* = 72)	Yes (*n* = 81)			
*Discharge treatment*						
Statin	110 (71.9)	49 (68.1)	61 (75.3)	0.32	1.66 (0.74–3.73)	0.22
Beta-blocker	51 (32.7)	20 (27.8)	30 (37.0)	0.22	1.39 (0.63–3.06)	0.42
Anxiolytic	16 (10.5)	5 (6.9)	11 (13.6)	0.18	1.41 (0.42–4.75)	0.58
Hypnotic	21 (13.7)	8 (11.1)	13 (16.1)	0.38	0.78 (0.25–2.45)	0.68
Antidepressant	20 (13.1)	5 (6.9)	15 (18.5)	0.034	2.13 (0.65–6.98)	0.21
Antihypertensive	110 (71.9)	51 (70.8)	59 (72.8)	0.78	1.17 (0.53–2.58)	0.70
Antidiabetes medications	17 (11.1)	5 (6.9)	12 (14.8)	0.12	1.64 (0.52–5.10)	0.40
Antiplatelet drug	122 (79.7)	59 (81.9)	63 (77.8)	0.52	1.09 (0.44–2.71)	0.85
Anticoagulant	36 (23.6)	16 (22.2)	20 (24.7)	0.72	0.93 (0.39–2.21)	0.86
Number of treatments, median (IQR)	3 (2–4)	3 (2-3)	3 (2–4)	0.024	1.30 (0.92–1.83)	0.14

*6-month treatment*						
Statin	116 (75.8)	53 (73.6)	63 (77.8)	0.55	1.89 (0.80–4.46)	0.15
Beta-blocker	51 (33.3)	19 (26.4)	32 (39.5)	0.086	1.73 (0.80–3.71)	0.16
Anxiolytic	20 (13.1)	8 (11.1)	12 (14.8)	0.50	0.75 (0.25–2.23)	0.61
Hypnotic	17 (11.1)	8 (11.1)	9 (11.1)	1.00	0.67 (0.20–2.20)	0.51
Antidepressant	26 (17.0)	6 (8.3)	20 (24.7)	0.007	2.08 (0.70–6.14)	0.18
Antihypertensive	124 (81.1)	55 (76.4)	69 (85.2)	0.17	1.92 (0.77–4.78)	0.16
Antidiabetes medications	18 (11.8)	6 (8.3)	12 (14.8)	0.21	1.64 (0.52–5.10)	0.40
Antiplatelet drug	105 (68.6)	51 (70.8)	54 (66.7)	0.58	1.21 (0.54–2.70)	0.64
Anticoagulant	50 (32.7)	21 (29.2)	29 (35.8)	0.38	0.95 (0.42–2.12)	0.90
Number of treatments, median (IQR)	3 (3-4)	3 (2–4)	3 (3-4)	0.020	1.34 (0.94–1.91)	0.11

Values are expressed as number (%) unless otherwise indicated.

^*∗*^Calculated using logistic regression model adjusted for gender, coronary heart disease history, 6-month Barthel Index score, depression, anxiety, and sleep disturbances (after multiple imputation to handle missing values on covariate).

**Table 3:** Comparison of medications at discharge and 6 months after stroke.

	Discharge, *n* (%)	6 months, *n* (%)	Discordance, *n* (%)	*p* value^*∗*^
Statin	110 (71.9)	116 (75.8)	16 (10.5)	0.13
Beta-blocker	50 (32.7)	51 (33.3)	15 (9.8)	0.80
Anxiolytic	16 (10.5)	20 (13.1)	20 (13.1)	0.37
Hypnotic	21 (13.7)	17 (11.1)	14 (9.2)	0.29
Antidepressant	20 (13.1)	26 (17.0)	16 (10.5)	0.13
Antihypertensive	110 (71.9)	124 (81.1)	14 (9.2)	<0.001
Antidiabetes medications	17 (11.1)	18 (11.8)	1 (0.7)	—
Antiplatelet drug	122 (79.7)	105 (68.6)	23 (15.0)	<0.001
Anticoagulant	36 (23.6)	50 (32.7)	20 (13.1)	0.002

^*∗*^Calculated using McNemar test.

**Table 4:** Association of the Chalder Fatigue Scale (CFS) score and medication use 6 months after stroke onset.

	Median (IQR) of CFS by use or nonuse of medications		*p* value^*∗*^	*p* value^†^
	No use	Use		
*6-month treatment*				
Statin	3 (1–6)	4 (1–6)	0.70	0.23
Beta-blocker	3 (1–6)	4 (2–7)	0.15	0.14
Anxiolytic	4 (1–6)	6 (2.5–9.5)	0.017	0.61
Hypnotic	4 (1–6)	4 (2–6)	0.76	0.66
Antidepressant	3 (1–6)	7 (5–9)	<0.001	0.059
Antihypertensive	1 (0–5)	4 (2–6)	0.033	0.072
Antidiabetes medications	4 (1–6)	4 (2–7)	0.28	0.36
Antiplatelet drug	4 (1–6)	4 (1–6)	0.97	0.23
Anticoagulant	4 (1–6)	4 (1–6)	0.98	0.24
Number of treatments		*ρ* = 0.27	<0.001	0.039

^*∗*^Unadjusted *p* values (Mann-Whitney *U* test or Spearman Rank Correlation (*ρ*) for number of treatments).

^†^Adjusted *p* values on gender, coronary heart disease history, 6-month Barthel Index (<100), depression score, anxiety score, and sleep disturbances (linear regression model on rank-transformed data after handling missing values on covariates by multiple imputation).
